# Glioblastoma in Elderly Patients: Current Management and Future Perspectives

**DOI:** 10.3390/cancers11030336

**Published:** 2019-03-08

**Authors:** Giuseppe Minniti, Giuseppe Lombardi, Sergio Paolini

**Affiliations:** 1 Radiation Oncology Unit, UPMC Hillman Cancer Center, San Pietro Hospital FBF, 00189 Rome, Italy; 2 Department of Oncology, Veneto Institute of Oncology IOV-IRCCS, 35128 Padua, Italy; giuseppe.lombardi@iov.veneto.it; 3 IRCCS Neuromed, 86077 Pozzilli (IS), Italy; nch@neuromed.it

**Keywords:** glioblastoma, elderly, surgery, radiotherapy, chemotherapy, temozolomide

## Abstract

The incidence of glioblastoma (GBM) in the elderly population is slowly increasing in Western countries. Current management includes surgery, radiation therapy (RT) and chemotherapy; however, survival is significantly worse than that observed in younger patients and the optimal treatment in terms of efficacy and safety remains a matter of debate. Surgical resection is often employed as initial treatment for elderly patients with GBM, although the survival benefit is modest. Better survival has been reported in elderly patients treated with RT compared with those receiving supportive care alone, with similar survival outcome for patients undergoing standard RT (60 Gy over 6 weeks) and hypofractionated RT (25–40 Gy in 5–15 daily fractions). Temozolomide, an alkylating agent, may represent an effective and safe therapy in patients with promoter methylation of O^6^-methylguanine-DNA-methyltransferase (*MGMT*) gene which is predictor of responsiveness to alkylating agents. An abbreviated course of RT, 40 Gy in 15 daily fractions in combination with adjuvant and concomitant temozolomide has emerged as an effective treatment for patients aged 65 years old or over with GBM. Results of the National Cancer Institute of Canada Clinical Trials Group (NCIC CTG CE6) and European Organization for Research and Treatment of Cancer (EORTC 26062/22061) randomized study of short-course RT with or without concurrent and adjuvant temozolomide have demonstrated a significant improvement in progression-free survival and overall survival for patients receiving RT and temozolomide over RT alone, without impairing either quality of life or functional status. Although combined chemoradiation has become the recommended treatment in fit elderly patients with GBM, several questions remain unanswered, including the survival impact of chemoradiation in patients with impaired neurological status, advanced age (>75–80 years old), or for those with severe comorbidities. In addition, the efficacy and safety of alternative therapeutic approaches according to the methylation status of the O^6^-methylguanine-DNA methyl-transferase (*MGMT*) gene promoter need to be explored in future trials.

## 1. Introduction

Gliomas account for almost 80% of all primary malignant brain tumors. Glioblastoma (GBM) is the most frequent histology and accounts for more than 50% of gliomas in all age groups, with an incidence rate among elderly patients of 70 years and older of 17.5 per 100,000 person-years, and a relative risk of 3–4 times compared with young adults [1,2,3]. Considering that the population of 65 years or older is expected to increase in the next two decades in USA, Canada, Australia, and Europe, this age group will account for the majority of GBM cases in these nations, representing an important aspect of public health.

Based on the results of the EORTC-NCIC CTG phase III study showing a significant improvement in the median survival for patients aged 18–70 years who received chemoradiation over radiation therapy (RT) alone from 12.1 to 14.6 months, and an improvement in 2-year survival from 10% to 26%, respectively, the standard of care for adult patients with GBM is represented by post-operative standard RT (60 Gy in 30 fractions) with concurrent and adjuvant temozolomide [4,5]. However, the majority of elderly patients with GBM are less likely to receive standard chemoradiation because aggressive combined approaches are associated with lower survival benefit and increased toxicity.

Elderly patients with GBM have historically been treated with standard or hypofractionated RT, with a reported median survival in the range of 5 to 9 months [6,7,8,9,10,11,12,13,14,15,16,17]. For elderly patients with O_6_-methylguanine-DNA methyl-transferase (*MGMT*) gene promoter methylation, the alkylating agent temozolomide has emerged as an effective treatment option associated with a survival benefit [18,19,20]. Published results from the EORTC (26981-22981)/NCIC CTG (CE.3) randomized study of short-course RT with or without concurrent and adjuvant temozolomide have made a significant contribution to the management of GBM [21] showing that the addition of temozolomide to short-course RT in patients 65 years of age or older with newly diagnosed GBM resulted in significantly longer survival compared to short-course RT alone. Although this regimen is likely to become the new standard of care in the elderly population with GBM, questions if all older patients may receive combined chemoradiation regardless of advanced age (>75–80 years old), impaired neurological status, presence of comorbidities, or molecular profiling of the tumor remain open.

The purpose of this review is to summarize the published literature on the efficacy of RT and chemotherapy given alone or in combination in elderly patients with GBM, and to address important issues such as the importance of molecular profiling in predicting response to treatments, the impact of treatments on quality of life and neurocognitive outcomes, and future research priorities for this population.

## 2. Methods

There is no generally agreed criterion for definition of “older people”. A cut-off of over 60 or 65 years is often used (http://www.who.int/healthinfo/survey/ageingdefnolder/en/). Most developed world countries have accepted the chronological age of 65 years as a definition of “elderly” or older person; however, in the developed countries, the most relevant geriatric syndromes (e.g., insomnia, urinary incontinence, severe hearing/vision problem, functional decline, fall and depressive disorder) are most common over 70–75 years. In order to include all published studies reporting on older patients with GBM, we have used an age cutoff of 60 years old for defining older patients in the current research.

A literature search was conducted in MEDLINE PubMed evaluating older people with GBM. The search focused on randomized, prospective and retrospective studies published in English. The literature search was performed using a combination of medical subject headings (MeSH) “glioblastoma” and free text terms (“radiation therapy” or “hypofractionated radiotherapy” or “chemotherapy” or “chemoradiation” or “elderly”). We included relevant studies published from 1990 to 2017. Studies published in languages other than English or not involving human subjects were not reviewed. A total of 178 potentially relevant studies were identified, including 19 prospective/randomized studies and 159 retrospective studies. The results of the literature research were used and included if appropriate.

## 3. Overview of Treatments

### 3.1. Surgery

Surgical resection is the first step in treating patients with GBM. The goals of the surgical procedure include histologic diagnosis, relief of the tumor mass effect, safe tumor cytoreduction, and possibly prolong patient survival. Although several reports have found that extensive surgical resection is associated with longer survival [22,23,24,25,26,27,28,29,30,31,32], aggressive surgery is still a controversial issue in neurosurgery. Simpson et al. [22] reviewed the impact of the extent of surgery on the survival outcome in 645 patients with GBM who were enrolled in three consecutive randomized Radiation Therapy Oncology Group (RTOG) trials. Patients undergoing gross total resection had a significant longer median survival than those receiving a biopsy only (11.3 vs. 6.6 months; *p* < 0.001); notably, a significant difference in median survival times was also found for partial resection compared with biopsy only treatment (10.4 vs. 6.6 months; *p* < 0.01). In a series of 788 patients who underwent surgery for a malignant glioma between 1997 and 2001 in North America, Laws et al. [23] showed that total/subtotal surgical resection was an independent favorable prognostic factor compared with biopsy only (*p* < 0.0001), with no difference between older and younger patients. In another series of 1229 patients aged less than 80 years with histologically verified GBM undergoing surgery at the University of Texas MD Anderson Cancer Center, Li et al. [32] reported a median survival time of 15.2 months after complete resection and 9.8 months after incomplete resection (*p* < 0.001). This survival advantage was achieved without an increased risk of overall or neurological postoperative deficits even after correcting for prognostic factors including age, Karnofsky Performance Scale (KPS) score, and preoperative contrast-enhancing tumor volume. The favorable impact of complete resection on survival has been confirmed in published series reporting the use of modern surgical techniques, such as intraoperative magnetic resonance imaging, intraoperative ultrasonography, and fluorescence-guided surgery [28,29,33]. In a series of 243 patients enrolled in a phase III trial assessing the efficacy of fluorescence-guided surgery with 5-aminolevulinic acid for resection of malignant gliomas, Stummer et al. [29] showed that patients who underwent complete resection survived longer than those who had incomplete resection (16.7 vs. 11.8 months, *p* < 0.0001), even in elderly patients older than 60 years of age with GBM.

A few studies have evaluated the efficacy of surgery in elderly patients with GBM [34,35,36,37,38,39,40]. In a systematic review and meta-analysis of biopsy versus partial versus gross total resection in patients older than 60 years of age with high-grade glioma, Almenawer et al. [37] have compared overall survival, KPS, progression-free survival, mortality, and morbidity amongst 12607 patients who were included in 34 studies. The overall survival was 5.71 months (95% CI 5.04–6.36) in patients undergoing biopsy, 8.68 months (95% CI 7.87–9.48) in those having subtotal resection, and 14.04 months (95% CI 12.8–15.2) in those subjected to gross total resection. For the entire population, patients undergoing overall resection (of any extent) had a significant benefit compared with those having biopsy, with mean difference in overall survival of 3.88 months (95% CI 2.14–5.62, *p* < 0.001). Mean difference in postoperative KPS was 10.4 (95% CI 6.58–14.22, *p* < 0.001) and mean difference in progression-free survival was 2.44 months (95% CI 1.45–3.43, *p* < 0.001). Overall, the analysis showed longer survival time, delayed tumor progression rate, and improved functional recovery with decreasing trends of mortality and morbidity rates in the order of biopsy, sub-total resection, and complete resection, suggesting a progressive improvement in clinical outcomes with greater degrees of resection. In a small randomized study of 30 patients older than 65 years with malignant glioma who received stereotactic biopsy or surgical resection, Vuorinen et al. [35] reported median survival times of 171 and 85 days after surgical resection or biopsy, respectively (*p* = 0.035). Similar results have been reported in other retrospective series showing significantly improved survival in patients with GBM receiving subtotal/complete resection compared with those undergoing stereotactic biopsy [36,37,38,39,40].

Biopsy, which has limited mortality and serious morbidity in the range of 1–5%, is usually performed to assess histological and molecular characteristics of the tumor when surgical resection carries high risks [41,42,43,44]. Tumor tissue is in fact fundamental to assess the molecular profile of GBMs and consequently to tailor the appropriate treatment. In this contest biopsy can be avoided only when clinical and radiological data provide an accurate diagnosis of GBM and results will not affect treatment choices.

Overall, findings from these studies support the general principle of considering maximal degrees of tumor removal when the operative option is indicated, regardless of age. The main limitation of these studies is a lack of detail about surgical complications, functional recovery time, and neurocognitive outcome following variable levels of resections. Preventing new permanent neurological deficits and maintaining good quality of life are essential factors for guiding surgical management of these patients. In absence of randomized studies, in current clinical practice the optimal surgical approach in elderly patients should be individually based on the carefully evaluation of known established safety measures, as those included in the neurologic assessment in neuro-oncology (NANO) scale [45] and in geriatric assessment models [46].

### 3.2. Radiotherapy

Postoperative RT, either standard RT or abbreviated courses of RT, has been historically employed for elderly patients with GBM [6,7,8,9,10,11,12,13,14,15,16,17]. A summary of randomized controlled trials addressing the efficacy and safety of RT in the elderly population with GBM is shown in Table 1 [14,15,17,19,20,40].

The superiority of RT (50 Gy in 28 daily fractions) over best supportive care has been demonstrated in a French multi-institutional randomized trial of 85 elderly patients with GBM aged 70 years or older with a KPS score of 70 or higher [47]. Median overall survival and progression-free survival times were 29.1 and 14.9 weeks for patients receiving RT plus supportive care and 16.9 and 5.4 weeks for those receiving supportive care alone, respectively. Notably, RT did not cause further deterioration in the KPS, health-related quality of life and cognitive functions compared with supportive care. The efficacy of RT versus supportive care alone has been demonstrated in other few studies [6,8,10,11,12,14,16,48]. Using the Surveillance, Epidemiology, and End Results (SEER) registry (1988–2004) as data source of 10987 patients with GBM aged 70 years or older, Scott and colleagues [41] showed that the overall survival time was significantly improved by RT after adjusting for surgery, tumor size, gender, ethnicity, and age at diagnosis. In another retrospective series of 202 patients with GBM treated between 1990 and 2000 at Leiden University, Marijnen et al. [16] reported a significant longer survival of 10.6 months in patients treated with RT compared with 1.9 months in non-irradiated patients (*p* < 0.0001), with no significant survival difference between elderly and young patients.

Few randomized studies have addressed the efficacy and safety of either radical RT or abbreviated courses of RT in elderly patients with GBM [15,17,19] (Table 1). In a randomized trial of 100 patients with GBM aged 60 years or older who received postoperative standard RT or short-course RT (40 Gy in 15 fractions over 3 weeks), Roa et al. [15] showed no survival differences between the two groups. Median survival times and 6-month survival rates were 5.1 months and 44.7% for patients treated with standard RT and 5.6 months and 41.7% for those receiving short-course RT, respectively.

The Nordic randomized, phase III trial enrolled 291 patients older than 60 years of age with newly diagnosed GBM who were assigned to receive three different treatments: temozolomide (200 mg/m^2^ on days 1–5 of every 28 days for up to six cycles), hypofractionated RT (34 Gy given in 3.4 Gy fractions over two weeks), or standard RT (60 Gy given in 2 Gy fractions over 6 weeks) [19]. The efficacy of radiation treatments was similar between the two radiation groups. The median survival time was 7.5 months for patients treated with hypofractionated RT and 6.0 months for those receiving standard RT; however, in patients older than 70 years hypofractionated RT resulted in significantly longer survival than standard RT (7.0 months vs. 5.2 months, *p* = 0.02).

Roa et al. [17] conducted a randomized trial of 98 frail and/or elderly patients aged 65 years and older with GBM who were randomized to receive two different hypofractionated radiation schedules. Median overall survival and progression-free survival times were 7.9 and 4.2 months in patients who received 25 Gy in five daily fractions and 6.4 and 4.2 months in those subjected to 40 Gy in 15 daily fractions over three weeks, respectively (*p* = 0.9). Neurological outcome and quality of life were similar between the two groups at 4 weeks and 8 weeks after treatment.

The assessment of neurocognitive status and quality of life is of particular relevance in elderly patients with GBM (Table 1). In Roa et al. [15] trial comparing standard RT versus short-course RT, KPS scores varied markedly over time but were not significantly different between groups; notably, 20% of elderly patients receiving standard RT interrupted the treatment because of acute toxicity. In the Nordic study, global health status and several functioning scales, including physical, role, emotional, social, functioning and cognitive (assessed by the European Organisation for Research and Treatment of Cancer (EORTC)) quality of life questionnaire Core 30 (QLQ-C30) did not change significantly between patients receiving standard or hypofractionated RT [19]. However, data need be interpreted with caution due to the low number of patients who completed questionnaires. A similar improvement of quality of life and performance status have been reported using hypofractionated schedules of 25–40 Gy in 5–15 daily fractions [6,8,9,14,17].

In summary, RT is associated with an improved survival in elderly patients with GBM with no significant detrimental effects on neurocognitive function and quality of life. Although standard RT may represent a feasible treatment option for elderly patients of 60–70 years old with a good performance status, results of randomized controlled studies comparing standard and hypofractionated radiation schedules clearly indicate that short-course RT should be recommended in elderly patients because it offers similar survival benefit and shortens the time of treatment. In addition, advanced radiation techniques, including intensity-modulated radiotherapy (IMRT), volumetric-modulated arc therapy (VMAT), and image-guided RT (IGRT), which allow the delivery of more precise radiation doses to the target while minimizing exposure of the surrounding normal brain tissues, should be routinely used in clinical practice to treat these patients with the objective to reduce the risk of neurocognitive deficits.

### 3.3. Chemotherapy

Historically, the role of chemotherapy as initial treatment for elderly patients with GBM has been poorly investigated in the past, mainly because of the concern about the limited efficacy [49,50] and the severity of side effects of nitrosurea-based regimens [51,52]. Recently, the use of temozolomide as an alternative to RT in older patients with malignant gliomas has been addressed in prospective and randomized studies (Table 1) [18,19,20,53,54].

The German Neuro-oncology Working Group (NOA) phase 3 trial (NOA-08) has compared the efficacy and safety of RT to temozolomide in elderly patients with anaplastic astrocytoma or GBM; 373 patients aged 65 years and older with histologically confirmed tumors, and a KPS score ≥ 60, were randomly assigned to receive dose-dense temozolomide (one week on, one week off cycles) or standard RT [20]. Median survival times and one-year overall survival rates were 8.6 months and 34.4% for patients receiving temozolomide, and 9.6 months and 37.4% for those treated with standard RT, respectively, indicating that chemotherapy was non-inferior to standard RT. Similarly, median event-free survival times (progression or death as event) were not different: 3.3 months for patients treated with temozolomide and 4.7 months for those treated with standard RT. The major novelty of the study was the strong predictive role of *MGMT* promoter methylation status on event-free survival outcome. Event-free survival rates were significantly better in patients with *MGMT* methylated promoter than in those with *MGMT* unmethylated promoter, although this favorable impact was seen only in patients receiving temozolomide. For patients with a methylated *MGMT* promoter, event-free survival rates were better with temozolomide than RT, while the opposite was true for patients with an unmethylated *MGMT* promoter. Analysis of health-related quality of life scales showed no significant differences between the two groups; however, grade 2–4 adverse events were more frequent in patients receiving temozolomide.

In the Nordic trial, elderly patients receiving temozolomide had better survival outcome than those having RT. The median survival time was 8.3 months after temozolomide and 6.0 months after standard RT (HR 0.7, 95% CI 0.52–0.93, *p* = 0.01); however, with no significant differences between patients receiving standard RT and hypofractionated RT (6.0 months vs. 7.5 months, HR 0.82, 95% CI 0.63–1.06, *p* = 0.12) [19]. As for the NOA-8, a striking finding of the study was the predictive value of *MGMT* promoter methylation status. For patients receiving temozolomide, the median survival was 9.7 months in patients with *MGMT* promoter methylation and 6.8 months in those without methylation (*p* = 0.02). In contrast, *MGMT* methylation status did not affect the survival in patients having RT (methylated tumors, 8.2 months; unmethylated tumors, 6.8 months; *p* = 0.81). EORTC QLQ-C30 data evaluating global health status and cognitive functioning were generally better in patients receiving temozolomide than those having standard RT.

A French phase II trial including 70 patients aged 70 years and older with newly diagnosed GBM and a postoperative KPS score < 70, showed that temozolomide alone, given at doses of 150 to 200 mg/m^2^/die for 5 days every 4 weeks until disease progression, resulted in improved functional status and quality of life, with a substantial proportion of patients who became capable of self-care, especially those with an *MGMT* methylated tumor [18]. The median and 6-month survival rates were 31 weeks and 69.2% in patients with *MGMT* promoter methylation and 18.7 weeks and 28% in those without methylation (*p* = 0.03), respectively. The same ANOCEF French group has recently published the results of another phase II trial exploring the combination of temozolomide and bevacizumab in 66 patients aged 70 years and older with a KPS < 70 and histologically confirmed GBM [54]. With a median overall survival (OS) of 23.9 weeks, cognition and quality of life significantly improved over time during treatment, and 33% of patients became transiently capable of self-care. Grade 3 or 4 hematologic toxicity and high blood pressure occurred in 20% of patients. Other toxicities, including venous thromboembolism, cerebral hemorrhage, and intestinal perforation were less common (<5%).

In summary, temozolomide is an effective and tolerated treatment for elderly patients with GBM associated with a significant improvement of functional status and quality of life. Response to treatment is significantly associated with *MGMT* promoter methylation status. For patients with *MGMT* methylated tumors, temozolomide results in longer compared with standard RT; in contrast, there is no evidence of a survival benefit in patients with *MGMT* unmethylated tumor. In clinical practice, this means that postoperative temozolomide should be considered only in patients with *MGMT* promoter methylated tumors, whereas its use is not recommended in those with *MGMT* unmethylated tumors.

### 3.4. Combined Chemoradiation

The use of standard or hypofractionated RT in combination with concomitant and/or adjuvant TMZ has been evaluated in several studies [55,56,57,58,59,60,61,62,63,64,65,66,67,68,69]. Results from published prospective series are shown in Table 2 [21,56,57,60,64].

In a small prospective series of 32 patients aged 70 years and older with a good KPS receiving standard RT with adjuvant and concomitant temozolomide at Sant’Andrea Hospital (University of Rome Sapienza, Rome, Italy) the median survival time and one-year survival rates were 10.6 months and 37%, respectively [56]; grade 3 or 4 hematologic toxic effects occurred in 24% of patients. In another prospective study of 58 patients aged 65 years and older with GBM treated with standard RT and concomitant and adjuvant temozolomide, Brandes et al. [57] observed a median survival of 13.7 months. *MGMT* methylation status was an independent prognostic factor for survival; the two-year survival rates were 83% for patients with methylated tumors and 56% for those with unmethylated tumors. Grade 3 or 4 hematological toxicity occurred in 10% of patients and grade 3 neurocognitive deterioration in 25% of patients. A similar incidence of neurological toxicity up to one third of elderly patients with GBM treated standard chemoradiation has been reported in few series [58,59].

The efficacy and safety of hypofractionated RT with or without temozolomide has been recently evaluated by the intergroup EORTC 26062-22061/NCIC CTG (CE.3) randomized trial comparing an abbreviated course of RT (40 Gy in 15 fractions) plus concomitant and adjuvant temozolomide versus abbreviated RT alone in 562 patients older than 65 years old with newly diagnosed GBM [21]. The median survival time (9.3 vs. 7.6 months, *p* < 0.0001) and progression-free survival time (5.3 vs. 3.9 months, *p* < 0.0001) were significantly better in patients receiving combined chemoradiation over RT alone. *MGMT* promoter methylation status was a strong predictor for survival. Amongst 165 patients with *MGMT* methylated promoter, the overall survival was 13.5 months in patients receiving RT and temozolomide and 7.7 months in those receiving RT alone (*p* = 0.0001); in patients with *MGMT* unmethylated promoter, the respective overall survival times were 10.0 months and 7.9 months (*p* = 0.055). Quality of life analysis assessed by the EORTC QLQ-C30 questionnaire and the EORTC brain module (QLQ-BN20) showed that changes in global health status, functioning and symptom scales were similar in the two groups, although nausea and constipation were worse in patients receiving temozolomide.

A similar survival benefit has been reported in a phase 2 trial of 70 patients aged 70 years and older with newly diagnosed GBM treated with the same regimen at the University of Rome [64]. For the whole population, the median overall survival time and 1-year survival rates were 12.4 months and 58%, respectively. According to the *MGMT* promoter methylation status, the 1-year and 2-year survival rates were 81% and 20% in patients with *MGMT* methylated tumors, and 32% and 0% in those with *MGMT* unmethylated tumors, respectively (*p* = 0.0001). The treatment was well tolerated and resulted in a significant improvement or stability in global health, social functioning, and cognitive functioning scores between baseline and 6-month follow-up.

In summary, combined chemoradiation represents an effective therapeutic strategy for elderly patients with GBM. Standard RT and concomitant and adjuvant temozolomide remain a feasible treatment in patients aged less than 70 years with good KPS, although treatment-related neurotoxicity leading to serious disability and worsening of the quality of life represents a major concern. Based on the results of the EORTC 26062-22061/NCIC CTG (CE.3) randomized trial, elderly patients aged 70 year and older with *MGMT* promoter methylation who are considered eligible for combined modality treatment should be offered a short-course RT, 40 Gy in 15 fractions, in combination with concomitant and adjuvant temozolomide. Elderly patients not considered candidates for combined chemoradiation should be treated with short-course RT or temozolomide based on *MGMT* promoter methylation status [70].

## 4. Future Perspectives

Combined chemoradiation remains a matter of concern in frail patients presenting with functional deficits, multiple comorbidities and geriatric syndromes, like gait imbalance, malnutrition, delirium, and incontinence that make them more vulnerable to treatment-related toxicities [71,72,73]. Identification of frail, vulnerable, or fit patients is essential for making more appropriate treatment decisions for elderly patients. A comprehensive geriatric assessment which includes the evaluation of functional status, cognitive function, nutritional status, comorbidities, polipharmacy, and socioeconomic status needs to be incorporated in future clinical trials with the aim of improving treatment outcome and reducing the risk of adverse events [74,75]. Several instruments may be used to assess the different domains of geriatric assessment, including performance status scales, daily activities, cognitive function, presence of comorbidities, psycological status, health and nutritional status, and socioeconomic status [46,74,76]. For daily clinical practice, several screening tests have been developed, including the abbreviated comprehensive geriatric assessment (aCGA) [77], the Groningen frailty indicator (GFI), as the G8 [78], and the vulnerable elders survey-13 (VES-13) [79] to help neurooncologists to guide cancer treatment decision-making and improve quality of life and functional independence of elderly patients with GBM. Based on geriatric assessment, patients with a higher frailty index score would receive less aggressive treatments; e.g., temozolomide or RT alone according to *MGMT* methylation promoter status.

Recently, experts from United States, Canada, and Europe have developed the Neurologic Assessment in Neuro-Oncology (NANO) scale which is an objective and simple tool for an accurate assessment of neurological function [45]. The NANO scale evaluates nine major domains of neurologic function that are most relevant to patients with supratentorial, infratentorial, and brainstem tumors, including gait, strength, upper extremity ataxia, sensation, visual fields, facial strength, language, level of consciousness, and behavior. It will provide an accurate neurological evaluation of elderly patients with GBM in both clinical trials and daily practice.

A novel treatment modality for patients with GBM is represented by the tumor-treating fields (TTFields) device (Optune^®^, Novocure Ltd., Novocure Inc, Israel) which is a portable, battery-operated device that generates TTFields. Results from a prospective phase 3 trial, EF-14, comparing TTFields plus temozolomide versus temozolomide alone after standard chemoradiation in patients with GBM has shown significant longer survival and clinical improvement in those having TTFields plus temozolomide [80]. Based on these results, Optune^®^ has received FDA approval for adult patients with newly diagnosed supratentorial glioblastoma, in addition to standard postoperative chemotherapy, or as monotherapy for the treatment of recurrent GBM.

For patient of all ages, surgery, radiation and systemic therapies have been employed for recurrent tumors, although standards of care are not well defined. Currently, there are no prospective studies evaluating the management of older patients with recurrent GBM. Outside of the context of clinical trials, systemic therapy with either lomustine or bevacizumab may represent a feasible treatment option for fit patients with recurrent tumors. Randomized studies evaluating the efficacy of lomustine given alone or in combination with other agents have observed a median survival in the range of 8–10 months [81,82,83]. In a phase III trial of 437 patients assigned to receive lomustine plus bevacizumab or lomustine alone, Wick et al. [83] showed a similar survival between the two groups (9.1 months versus 8.6 months; *p* = 0.65). Grade 3 to 5 adverse events occurred in 63.6% of the patients in the combination group and 38.1% of the patients in the monotherapy group; this means that the use of lomustine should be carefully considered in older and frail patients at increased risk of toxicity. The antiangiogenic agent bevacizumab has been approved at recurrence in various countries, but not in the European Union. Although bevacizumab did not have superior efficacy compared with lomustine [82,83], it produces evident symptom relief and steroid-sparing effects. Future clinical trials need to evaluate the efficacy and safety of different treatment approaches in elderly patients with recurrent GBM and good functional status.

Although data from EORTC 26062-22061/NCIC CTG (CE.3) randomized trial support the combination of hypofractionated RT with temozolomide in patients older than 65 years of age, the survival benefit in patients with unmethylated tumors remains questionable. In addition, no trials have compared the survival and neurocognitive outcomes of combined chemoradiation versus temozolomide alone in patients with *MGMT* promoter methylation. Thus, future trials are needed to explore these important issues. Finally, molecular profiling contributes to identify prognostic subgroups of elderly patients with GBM who may benefit from new therapies. Although recent trials have failed to demonstrate the efficacy of new agents in patients with GBM, including bevacizumab and cilengitide, the discovery of targeted agents and immunotherapy strategies which can translate to a survival benefit in patients with GBM remain an area of continued research.

## 5. Conclusions

For elderly patients with newly diagnosed GBM, current management includes surgery, RT and chemotherapy; however, survival is significantly worse than that observed in younger patients. Standard RT with concomitant and adjuvant temozolomide, which represent the standard of care for newly diagnosed adult patients with GBM in good general and neurological condition, may be considered in selected fit patients aged between 65 and 70 years. Elderly patients aged 70 years and older who are considered eligible for combined modality treatment should receive a short-course RT with concomitant and adjuvant temozolomide up to 12 cycles. In the absence of comparative data between temozolomide alone and chemoradiation, elderly patients with *MGMT* promoter methylation may be considered for temozolomide alone, especially those presenting with functional impairment and geriatric syndromes. Temozolomide alone is not associated with survival advantages in elderly patients with unmethylated tumors; patients who are not considered eligible for combined chemoradiation should receive hypofractionated RT. Supportive and palliative care may represent an appropriate approach for frail patients with large or multifocal tumors and low KPS at an increased of treatment-related toxicity.

## Figures and Tables

**Table 1:** Selected prospective studies on radiotherapy or chemotherapy in older patients with glioblastoma.

Authors	Type of Study	pts	Age Yrs	RT Dose Gy/fr	CHT	Median PFS Months	Median OS Months	Toxicity	Neurological Outcome and Quality of Life (QoL)
McAleese JJ et al., 2003 [14]	Prospective	30	65–70	30/6	no	NR	6 m 37%	Neurological deterioration. Occurred in 3% of patients.	68% of patients improved or remained stable, as assessed by Barthel score.
		29	≥70	30/6	no	NR	6 m 41%		
Chinot O et al., 2004 [53]	Prospective	32	≥70	no	TMZ*	5	6.4	Any grade 3–4 hematological toxicity 15%.	NR
						1-yr 15%	1-yr 25%		
Roa W et al., 2004 [15]	Randomized	51	≥60	60/30	no	NR	5.1	26% of patients receiving standard RT and 10% receiving short course RT discontinued RT for clinical deterioration.	No significant differences in KPS scores between groups; insufficient number of completed questionnaires for QoL evaluation.
		49	≥60	40/15	no	NR	5.6		
Keime-Guiber F et al., 2007 [47]	Randomized	39	≥70	50/28	no	3.6	7	No grade 3–4 toxicity reported.	QoL (QLQ-BN20) and neurological function by mini-mental state examination (MMSE) showed no differences between groups.
		39	≥70	no	no	1.5	4		
Gallego Perez-Larraya et al., 2011 [18]	Prospective	70	≥70	no	TMZ*	4	6	Any grade 3–4 hematological toxicities 25%.	33% of patients improved their KPS by 10 or more points, and 18 (26%) became capable of self-care (KPS ≥ 70). MMSE and QLQ C30-BN20 improved.
						1-yr 6.5%	1-yr 11.4%		
Malmstrom et al., 2012 [19]	Randomized	100	>60	60/30	no	NA	6 (1-yr 17%)	72% completed standard RT and 95% hypofractionated RT; Grade 3–4 hematological toxicity in 19% of patients receiving TMZ.	Global health status between groups; better cognitive and phisical functioning in TMZ group at 3 months (QLQC30-BN20).
		98	>60	34/10	no	NA	7.5 (1-yr 23%)		
		93	>60	no	TMZ*	NA	8.3 (1-yr 27%)		
Wick et al., 2012 [20]	Randomized	178*	>65	60/30	no	4.7 (1-yr 9.3%)	9.6 (1-yr 37.4%)	Grade 2–4 toxicities were more frequent in TMZ than RT group in all categories except for cutaneous adverse events.	QoL scales were siilar between groups (QLQC30-BN20), except for communication deficits, greater in RT group.
		195*	>65	no	TMZ^+^	3.3 (1-yr 12%)	8.6 (1-yr 34.4%)		
Roa et al., 2015 [17]	Randomized	48*	≥65	40/15	no	4.2	7.9	No grade 3–4 acute toxicity.	Similar mean global QoL scores at 8 weeks.
		50*	≥65	25/5	no	4.2	6.4		
Reyes-Botero, 2018 [54]	Prospective	66	≥70	no	TMZ* + Bev	4 months	5.8 months	Grade ≥ 3 hematological toxicity 20%, high blood pressure 24%, venous thromboembolism 4.5%, cerebral hemorrhage 3%.	Twenty-two (33%) patients became transiently capable of self-care (i.e., KPS > 70). Cognition and quality of life significantly improved over time during treament.

RT, radiotherapy; CHT, chemotherapy; OS, overall survival; PFS, progression-free survival; NR, not reported. TMZ, Temozolomide; *TMZ (200 mg/m^2^ on days 1–5) every 4 weeks; ^+^TMZ (200 mg/m^2^ 1 week on/1 week off); Bev, bevacizumab.

**Table 2:** Selected studies on combined radiochemotherapy in older patients with glioblastoma.

Authors	Type	Pts	Age	RT Dose	CHT	Median PFS	Median OS	Toxicity	Neurological Outcomeand Quality of Life (QoL)
	of Study		yrs	Gy/fr		Months	Months		
Minniti G et al., 2008 [56]	Prospective	32	≥70	60/30	TMZ	6.7 (1-yr 16%)	10.8 (1-yr 7%)	Neurological deterioration in 40%; grade 3–4 hematological toxicity 24%.	NR
Brandes et al., 2009 [57]	Prospective	58	≥65	60/30	TMZ	9.5 (1-yr 35%)	13.7 (2-yr 31.4%)	Grade 2 neurological deterioration, 31%; grade 3, 25%; grade 3–4 hematological toxicity, 9%.	NR
Minniti et al., 2009 [60]	Prospective	43	≥70	30/6	TMZ	6.3 (1-yr 12%)	9.3 (1-yr 35%)	Neurological deterioration in 16%; Grade 3–4 hematological toxicity 27%.	No significant decline in functioning scales and global health status (QLQC30-BN20) in patients free of disease progression.
Minniti et al., 2012 [64]	Prospective	70	≥70	40/15	TMZ	6 (1-yr 20%)	12.4 (1-yr 58%)	Grade 2/3 neurological toxicity, 10%; Grade 3–4 hematological toxicity, 29%.	Global health, social and cognitive functioning, and motor dysfunction improved over time (QLQC30-BN20); MMSE score improved or remained stable in 89% of patients free of disease progression.
Perry et al., 2016 [21]	Randomized	178*	>65	40/15	no	4.7 (1-yr 9.3%)	9.6 (1-yr 37.4%)	Grade 3–4 hematological toxicity in 25% and 9% of patients receiving RT plus TMZ or RT alone, respectively.	Changes from baseline scores during treatment and follow-up were similar by groups (QLQC30-BN20), with the exception of nausea and vomiting being worse in the RT + TMZ group.
		195*	>65	40/15	TMZ	3.3 (1-yr 12%)	8.6 (1-yr 34.4%)		

RT, radiotherapy; CHT, chemotherapy; OS, overall survival; PFS, progression-free survival; NR, not repordedTMZ, temozolomide given concomitantly (75 mg/m^2^/day) and adjuvantly (200 mg/m^2^ on days 1–5 every four weeks).
